# SPARKLE: a new spark in treating oligorecurrent prostate cancer: adding systemic treatment to stereotactic body radiotherapy or metastasectomy: key to long-lasting event-free survival?

**DOI:** 10.1186/s12885-022-10374-0

**Published:** 2022-12-12

**Authors:** Kato Rans, Berghen Charlien, Ameye Filip, De Hertogh Olivier, den Hartog Julie, Draulans Céderic, Dumez Herlinde, Engels Benedikt, Goffin Karolien, Laenen Annouschka, Liefhooghe Nick, Poels Kenneth, Salembier Carl, Slabbaert Koen, Vandendriessche Hans, Vanneste Ben, Joniau Steven, De Meerleer Gert

**Affiliations:** 1 Department of Radiation Oncology, University Hospitals Leuven, Herestraat 49, 3000 Leuven, Belgium; 2 Department of Urology, AZ Maria Middelares Ghent, Ghent, Belgium; 3 Department of Radiotherapy, Centre Hospitalier Régional de Verviers, Verviers, Belgium; 4 Department of General Medical Oncology, University Hospitals Leuven, Leuven Cancer Institute, Leuven, Belgium; 5 Department of Radiation Oncology, AZ Delta Roeselare-Menen-Torhout, Roeselare, Belgium; 6 Department of Nuclear Medicine and Molecular Imaging, University Hospitals Leuven, Leuven, Belgium; 7 Leuven Biostatistics and Statistical Bioinformatics Center, KU Leuven, Leuven, Belgium; 8 Department of Radiation Oncology, AZ Groeninge, Kortrijk, Belgium; 9 Department of Radiotherapy, Europe Hospitals Brussels, Brussels, Belgium; 10 Department of Urology, RZ Tienen, Tienen, Belgium; 11 Department of Urology, AZ Jan Portaels, Vilvoorde, Belgium; 12 Department of Human Structure and Repair; Department of Radiation Oncology, Ghent University Hospital, Ghent, Belgium; 13 Department of Urology, University Hospitals Leuven, Leuven, Belgium

**Keywords:** Hormone sensitive prostate cancer, Metastasis-directed therapy, Stereotactic body radiation therapy, Androgen deprivation therapy, Androgen receptor targeted therapy, Oligorecurrent prostate cancer, Metastatic prostate cancer

## Abstract

**Background:**

Metastasis-directed therapy (MDT) significantly delays the initiation of palliative androgen deprivation therapy (pADT) in patients with oligorecurrent prostate cancer (PCa) with a positive impact on patient’s quality of life. However, it remains unclear whether the addition of ADT improves polymetastatic free survival (PMFS) and metastatic castration refractory PCa-free survival (mCRPC-FS) and how long concomitant hormone therapy should be given. A significant overall survival (OS) benefit was shown when an androgen receptor targeted agent (ARTA) was added to pADT in patients with metastatic hormone sensitive PCa (HSPC). However, whether the addition of and ARTA to MDT in the treatment of oligorecurrent PCa results in better PMFS and mCRPC-FS has not been proven yet.

**Methods & design:**

Patients diagnosed with oligorecurrent HSPC (defined as a maximum of 5 extracranial metastases on PSMA PET-CT) will be randomized in a 1:1:1 allocation ratio between arm A: MDT alone, arm B: MDT with 1 month ADT, or arm C: MDT with 6 months ADT together with ARTA (enzalutamide 4 × 40 mg daily) for 6 months. Patients will be stratified by PSA doubling time (≤ 3 vs. > 3 months), number of metastases (1 vs. > 1) and initial localization of metastases (M1a vs. M1b and/or M1c). The primary endpoint is PMFS, and the secondary endpoints include mCRPC-FS, biochemical relapse-free survival (bRFS), clinical progression free survival (cPFS), cancer specific survival (CSS), overall survival (OS), quality of life (QOL) and toxicity.

**Discussion:**

This is the first prospective multicentre randomized phase III trial that investigates whether the addition of short-term ADT during 1 month or short-term ADT during 6 months together with an ARTA to MDT significantly prolongs PMFS and/or mCRPC-FS.

**Trial registration:**

ClinicalTrials.gov Identifier: NCT05352178, registered April 28, 2022.

## Background

### Metastasis-directed therapy

Up to 50% of patients treated with curative intent for high-risk prostate cancer (PCa) will eventually develop biochemical recurrence (BCR) at long-term follow-up [1, 2]. BCR is a precursor for the development of distant metastases and a predictor of PCa-related death. With the introduction of more sensitive imaging modalities such as prostate-specific membrane antigen (PSMA) positron emission tomography/computed tomography (PET/CT) or positron emission tomography/magnetic resonance imaging (PET/MRI) at the time of BCR, more patients are currently diagnosed with a limited number of metastases, a disease state defined as oligometastatic and in general pragmatically defined as the development of maximally 5 metastatic spots at one point in time [3]. More specific, in this setting, this disease status has been termed oligorecurrent PCa and has a different prognosis in comparison to polymetastatic PCa. Indeed, it has been demonstrated that the number of metastases is an important prognostic factor for PCa-specific survival (PCSS) [4–6]. The optimal treatment strategy in patients presenting with oligorecurrent PCa is still a matter of debate [7, 8]. While historically, palliative androgen-deprivation (pADT) therapy has been considered a state-of-the-art treatment, the advent of metastasis-directed therapy (MDT), performed by either metastasectomy or stereotactic body radiation therapy (SBRT) has broadened the therapeutic window as it significantly delays the initiation of pADT and its substantial side-effects when compared to active surveillance [9]. Retrospective studies served as a solid back-up demonstrating that the initiation of pADT could be delayed for several years which positively impacts patient’s quality-of-life (QOL) [9, 10]. Moreover, in some patients, the early use of pADT might promote the development of the castrate-resistant state, in which metastatic progression is driven by androgen-independent pathways [11].

Importantly, all the above-referred studies demonstrated that MDT using SBRT in case of oligorecurrent prostate cancer has low toxicity [9, 12, 13].

The SABR-COMET study [14] demonstrated that (for different tumor types, 21% of them being PCa patients) adding SBRT on top of the palliative systemic treatment in patients with a low-volume metastatic recurrence after previous treatment of the primary tumour significantly improved overall survival (OS) when compared to palliative systemic treatment alone. The eight-year OS was 27.2% in the SABR arm vs. 13.6% in the control arm (HR 0.5, 95% CI 0.3–0.84, *p* = 0.008) [15].

Our own research team published long-term outcomes of one of the largest retrospective analyses on MDT for oligorecurrent PCa after radical prostatectomy. In total, 191 oligorecurrent PCa patients were analysed. Estimated median palliative-ADT free survival was 66 months and estimated median mCRPC-free survival was not reached, but 83% of patients were still free of mCRPC at 10 years. In total, 314 MDTs were performed, and 25 patients (13%) received ≥ 3 MDTs. Repeated MDT resulted in low toxicity and could be offered to well-informed and motivated patients [13].

### Combining MDT with ADT

ADT has a radio-sensitizing effect by inhibiting the androgen receptor-mediated repair of radiotherapy (RT)-induced DNA damage in the PCa cells [16]. The addition of a non-timeless period of ADT to primary RT has shown to improve OS for intermediate- and high-risk PCa [17, 18]. Two recent trials also suggested a progression-free by adding temporary ADT to salvage RT, especially among young and fit patients [19, 20]. Vice-versa, the addition of RT to ADT improved OS in patients presenting with high-risk disease and patients presenting with pelvic nodal disease [21–25].

When SBRT for oligorecurrent disease is applied, it is not clear whether adding ADT for a certain period will extend the postponement of polymetastatic disease and the development of mCRPC. Scientific reports on MDT in PCa so far use SBRT as monotherapy or in combination with a certain period of ADT [12, 13, 26, 27].

### Combining ADT with second-line hormonal therapy

In three large randomized controlled trials (ENZAMET [28], ARCHES [29] and TITAN [30]), the addition of an androgen receptor targeted agent (ARTA) to ADT in men with metastatic hormone-sensitive PCa (mHSPC) was tested [28, 29, 31]. In ARCHES, the primary endpoint was radiographic progression-free survival (rPFS). rPFS was significantly improved when enzalutamide was added to ADT with a HR of 0.39 (0.3–0.5; *p* < 0.001). Approximately 36% of patients included in this trial had low-volume metastatic disease [29]. Recently the analysis of the secondary endpoints was published and concluded that the combination of enzalutamide with ADT reduced risk of death by 34% with a HR of 0.66 (0.53–0.81; *p* < 0.001) [32]. In the ENZAMET trial, the primary endpoint was overall survival (OS). The addition of enzalutamide to ADT improved OS with a HR of 0.67 (0.52–0.86; *p* = 0.002). Approximately half of the patients had low volume metastatic disease [28]. In the TITAN trial, apalutamide was added to ADT. The endpoints were rPFS and OS. rPFS was significantly improved in the combination group with a HR of 0.48 (0.39–0.60; *p* < 0.001). OS at 24 months was improved for the combination with a HR of 0.67 (0.51–0.89; *p* < 0.005). In this trial, 37% of patients had low volume metastatic disease [31]. In summary, all these trials resulted in the same conclusion: the addition of ARTA to ADT significantly improves clinical outcomes.

In two other large randomized controlled trials (STAMPEDE [33] and LATITUDE [34]), the addition of abiraterone acetate plus prednisone to ADT in men with mHSPC at first presentation was studied. Both trials showed a significant improvement in OS with a HR of 0.61 (0.53–0.70; *p* < 0.001) in the subgroup analysis for mHSPC in the STAMPEDE trial and a HR of 0.62 (0.51–0.76; *p* < 0,001) in the LATITUDE trial [33, 34]. Finally, the androgen receptor inhibitor, darolutamide, was tested in the ARASENS trial [35] in combination with ADT, and docetaxel among patients with mHSPC with OS as primary endpoint. The OS was significantly improved in the combination group with darolutamide with a HR of 0.68 (0.57–0.80; *p* < 0,001) [35].

But, although a substantial number of patients in the above-mentioned studies had low-volume metastatic disease, none of them examined the combination treatment of SBRT along with ADT and ARTA.

## Methods/design

### Study design

**Fig. 1:**
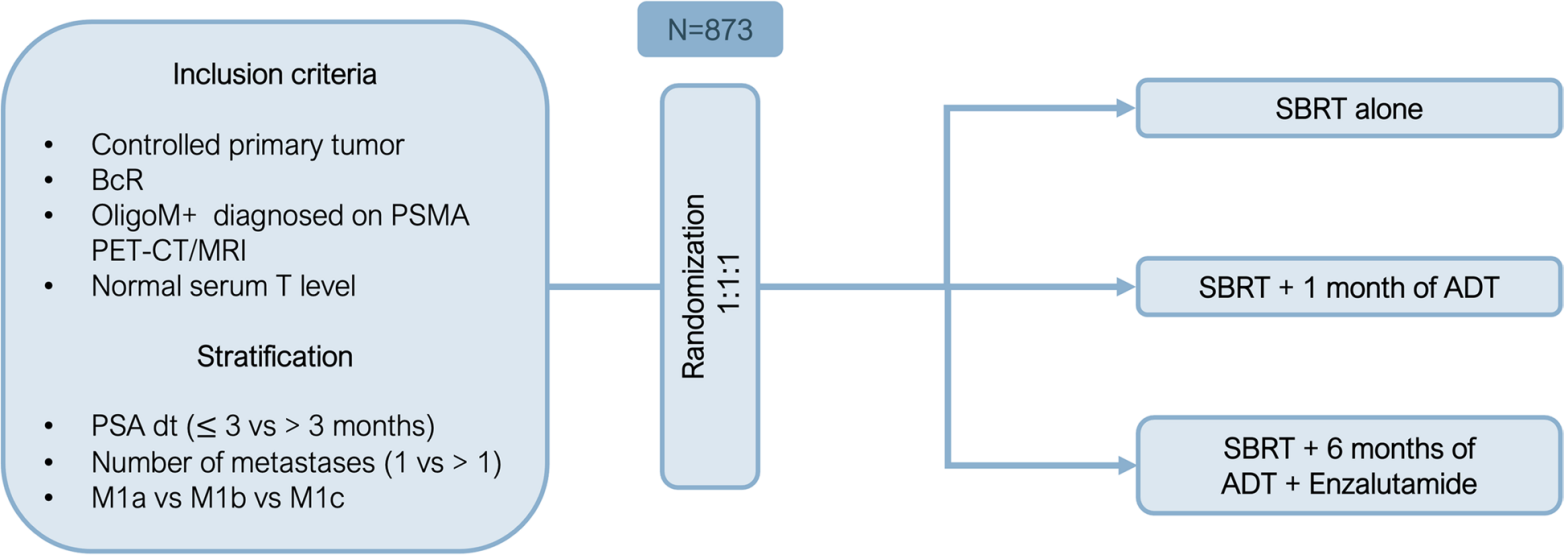
Trial design BcR: Biochemical recurrence; OligoM + : oligometastatic disease; PSMA PET-CT/MRI: prostate-specific membrane antigen (PSMA) positron emission tomography/computed tomography (PET/CT) or positron emission tomography/magnetic resonance imaging (PET/MRI); T: testosterone; PSA: prostate specific antigen; M1a: non-regional lymph node metastases, M1b: bone metastases, M1c: visceral metastases; SBRT: stereotactic body radiotherapy; ADT: androgen deprivation therapy

SPARKLE (a new **S**park in treating oligorecurrent **P**rostate cancer: **A**dding systemic treatment to stereotactic body **R**adiotherapy or metastasectomy: **K**ey to **L**ong-lasting **E**vent-free survival?) is approved by the Ethics committee of the Leuven University Hospitals (LUH) (EC2022/S65935) and is registered at the EudraCT data base (2022–000,066-18) and at clinicaltrials.gov (NCT05352178). It is a prospective, multicenter, randomized, open-label, three-arm, phase III study. The aim is to investigate whether the addition of short-term ADT during 1 month or short-term ADT during 6 months together with an ARTA to MDT significantly prologs polymetastatic-free survival (PMFS). The following arms will be compared (1:1:1) (Fig. 1):

- 1. Arm A: MDT alone
- 2. Arm B: MDT + 1 month of ADT

- 3. Arm C: MDT + 6 months of ADT together with 6 months ARTA (enzalutamide)

The primary endpoint is polymetastatic-free survival (PMFS).

### Inclusion criteria

All patients must fulfil the following inclusion criteria:

• Histologically proven initial diagnosis of prostatic adenocarcinoma.
• Prior treated and controlled primary tumor. Patients with local recurrence can be included only when accompanied by M1a-c disease, provided that the total number of active lesions does not exceed 5.
• Biochemical recurrence defined by prostate-specific antigen (PSA) values > 0.2 ng/ml (i.e., two consecutive increases) following radical prostatectomy ± postoperative RT and a PSA value of 2 ng/ml above the nadir after high-dose RT and confirmed at least once [36].
• Presence of oligorecurrent disease defined as a maximum of 5 extracranial metastases in any organ, diagnosed on PSMA PET-CT (PSMA PET-MRI is allowed, but optional) reported according to the E-PSMA consensus guidelines for interpretation of PSMA-PET [37]. Nodal (N1) disease can be included only when accompanied by M1a-c disease, provided that the total number of active lesions does not exceed 5.
• Serum testosterone level within normal range.
• WHO performance 0–2.
• Age >  = 18 years old.
• Willing to provide a signed informed consent.
• Absence of psychological, sociological, or geographical condition potentially hampering compliance with study protocol.
• Patients must be presented at the multidisciplinary board meeting and the inclusion in the trial needs approval by this board.

### Exclusion criteria

• Serum testosterone level at castration level.
• PSA rise while on active treatment (LHRH-agonist, LHRH antagonist, anti-androgen, maximal androgen blockade, oestrogens).
• Presence of poly-metastatic disease, defined as more than 5 extracranial metastatic lesions visible on PSMA PET-CT.
• Pelvic nodal disease (N1) without the presence of metastatic disease (M1a-c).
• Active malignancy other than prostate cancer that could potentially interfere with the interpretation of this trial (decision left to the principal investigator).
• Previous treatments (RT, surgery) or comorbidities rendering new treatment with SBRT or metastasectomy impossible.
• Contraindications for intake of enzalutamide (seizure or any condition that may predispose to seizure; significant cardiovascular disease within the last three months including myocardial infarction, unstable angina, congestive heart failure, ongoing arrythmias of grade > 2 or a thromboembolic event).
• Not able to understand the treatment protocol or sign the informed consent.

### Evaluation and randomization

Patients must be staged with PSMA PET-CT/MRI within 4 weeks prior to randomization. The study will employ a 1:1:1 randomization between arm A: arm B: arm C. Patients will be stratified according to PSA doubling time (≤ 3 vs. > 3 months), number of metastases (1 versus > 1) and initial localization of metastases (M1a vs. M1b and/or M1c) (Fig. 1). Randomization will be performed centrally at the University Hospitals Leuven.

### Interventions

**Fig. 2:**
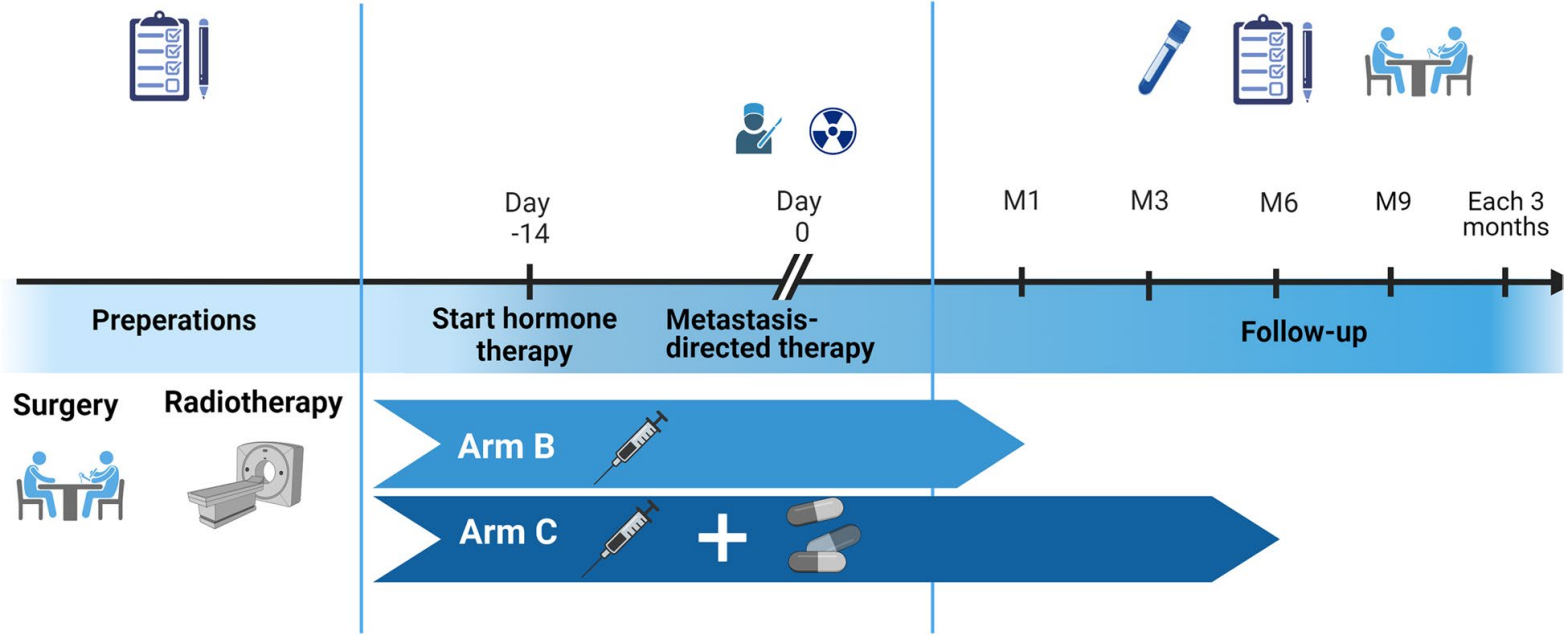
Participants timeline. Timeline depending on study arm and follow-up schedule

All patients eligible for the trial will be recruited after a mandatory discussion on the multidisciplinary urology tumour board, and after approval from the attending physician if a patients is referred from another external treatment centre. Leuven University Hospitals has started recruitment in April 2022. Participating treatment centres will be added subsequently following the legal ethical requirements i.e. starting at least 3 months after inclusion of the first patient in Leuven University Hospitals. After being considered eligible, the patient will follow the trial procedure. At first, a screen visit is scheduled, on which the patient will receive a standard clinical examination and laboratory analysis including peripheral blood cell count with formula, liver and kidney function, alkaline phosphatase, lactate dehydrogenase, PSA and testosterone. Three Quality-of-life questionnaire (QLQ-30, QLQ-PR25 and EQ-5D) [38, 39] as well as a toxicity score will be carried out [40] after signature of the informed consent. Post-treatment follow-up will be planned according to the trial protocol: there are 5 follow-up visits (month 1 and further on 3-monthly basis) scheduled in year 1 and 3-monthly follow-up visits thereafter unless clinical evolution requires more monitoring. The timeline of the participants is shown in Fig. 2. The summary of the study visits is displayers in Table 1.

**Table 1:** Trial flowchart

Preliminary investigations	Radiotherapy:	
	‐ CT-simulation	
	‐ MRI for radiotherapy planning	
	‐ **Completing the questionnaire**	
	Surgery:	
	‐ Pre-operative consultation anesthesia	
	‐ **Completing the questionnaire**	
Start hormone therapy (14 days before the metastasis-directed therapy)	Group A	/
	Group B	start hormone therapy for 1 month
	Group C	start first-line hormone therapy + second-line hormone therapy for 6 months
Metastasis-directed therapy	- Surgery - Radiotherapy: 3–7 irradiation treatments, max 3x/week	
End of Radiotherapy	- Standard follow-up + blood sample + **completing the questionnaire**	
Month 1	- Standard follow-up + blood sample + **completing the questionnaire**	
Month 3		
Month 6		
Month 9 and every 3 months thereafter	- Standard follow-up + blood sample + **completing the questionnaire at month 12 and 24**	

Summary of study visits and procedures

### Stereotactic body radiotherapy

For the treatment with SBRT, all patients will receive a CT simulation with 1 mm CT slice thickness through the oligorecurrence site(s) and neighbouring organs at risk. In case of non-bony oligorecurrence, intravenous contrast will be applied (standard procedure). For lung lesions we recommend motion management with a 4D-CT simulation scan. The isocenter will be placed in the center of the oligorecurrent lesion (standard procedure). In order to increase patient comfort and stability, different support devices can be used depending on the location of the metastases and following the standard procedure of the participating centres. Delineation of the oligorecurrent metastatic lesion(s) as gross target volume (GTV) will be based on CT and hybrid PSMA PET imaging (PET-CT/MRI). The additional use of magnetic resonance imaging is left at the discretion of the treating team, however, is highly recommended in case of bony metastases. The GTV will be expanded to create the Planning Target Volume (PTV) with a margin that accounts for setup error and organ motion with a maximum of 7 mm. Using intensity-modulated arc therapy (IMAT) or volumetric arc therapy (VMAT), the aim is to prescribe a biologically equivalent dose (BED) of at least 100 Gy using an alpha over beta of 3. Typically, a total dose of 36 Gy will be prescribed in 3 fractions of 12 Gy. Treatment will be prescribed to the periphery of the target and (80% of the dose should cover 90% of the PTV). Dose constraints for organ at risk (OAR) will be in accordance the AAPM task group 100 report [41]. Dose constraints for OAR take precedence over the prescribed dose to the PTV. Treatment will be delivered using photons from a linear accelerator. Image-guided RT is mandatory and will be performed using daily cone-beam CT. Fractions will be separated > / = 48 h and < 72 h.

### Surgery

The surgical technique to be used is at the discretion and expertise of the surgeon but has to be in accordance with the best surgical practice available. When possible, minimally invasive techniques are preferred. A template-based lymph node dissection (LND) is preferred above removing only the suspicious node in the case of lymph node metastasis, but not mandatory. Pelvic LND is defined as the removal of lymph nodes distal to the aortic bifurcation (internal, external and obturator iliac, common iliac, perirectal and/or presacral). Retroperitoneal LND is defined as the removal of lymph nodes above the aortic bifurcation, with upper level at least 1 cm above the most cranial lesion. The surgical removal of bone metastasis or visceral metastasis will always be performed in consultation and according to the expertise of the surgeon specialized for the respective location of metastasis.

### Hormonal therapy

Androgen deprivation therapy (goserelin, leuprorelin, triptorelin, degarelix) has to be started at least 2 weeks before the RT. SBRT must be completed within the timeframe of chemical castration i.e. within 4 weeks in Arm B and within 6 months in Arm C. To prevent flare-up, bicalutamide 50 mg will also be started once daily for 1 month in case of administration of goserelin, leuprorelin or triptorelin. From the second week of bicalutamide intake, ADT can be initiated.

### Enzalutamide

Enzalutamide (Xtandi®, Astellas Pharma) has to be started together with the start of ADT.

### Premature discontinuation of trial treatment

Participants may voluntary withdraw their consent to participate in the Trial for any reason at any time. The participant’s request to withdraw from the Trial must always be respected without prejudice or effect on further treatment. Withdrawal of consent will be documented in the participant’s medical record. Trial data and samples collected before withdrawal can be used in the trial. No new trial data or samples will be collected after withdrawal of the participant. The investigator may also decide at any time during the course of the study to temporarily suspend or permanently discontinue the Trial treatment if continuation of the study would be detrimental to, or not in the best interest of the participant. Similarly, the Sponsor, Ethics Committee or authorized regulatory authority may decide to discontinue or prematurely terminate the Trial when new information becomes available whereby the rights, safety and well-being of Trial participants can no longer be assured, when the integrity of the Trial has been compromised, or when the scientific value of the Trial is outdated and/or unjustifiable. In any case of premature termination of the study and/or interruption/discontinuation of treatment, the Investigator will continue to monitor the participant’s condition and provide adequate medical care and follow-up. In all situations where study participation is discontinued, as well as when the study is completed as planned, the investigator will assess the participants health and prescribe the best treatment available at that time.

### Objectives

#### Primary endpoint

- ◦ Poly-Metastatic Free Survival (PMFS) will be determined from the last day of the first SBRT or from the day surgery was performed until the first day of poly-progression which is defined as the detection > 5 new lesions at PSMA PET-CT/MRI (± combined with MRI if needed to improve diagnostic accuracy). In case of poly-progression, pADT combined with chemotherapy [42–44], ARTA [28, 29, 31] or abiraterone-acetate [33, 34] will be considered the standard-of-care. The decision on which treatment will be offered will be taken on the multidisciplinary meeting in every case and will take into account former treatment. If recurrence occurs in ≤ 5 lesions, a new MDT is proposed if technically feasible and the patient will receive again the treatment he was allocated to at the time of initial randomisation. Patients free from poly-progression are censored at their last follow-up.

#### Secondary endpoints

- ◦ Metastatic Castration-Refractory Prostate Cancer Free Survival (mCRPC-FS) will be determined from the last day of the first SBRT or from the day surgery was performed until the first day of diagnosis of CRPC. CRPC is defined according to the contemporary EAU-guidelines as the time to biochemical and/or clinical progression at castrate testosterone levels (< 50 ng/dl). Biochemical progression is defined as three consecutive PSA rises (1 week interval), of which at least 2 rises with a PSA level of > 2 ng/ml and a rise of 50% above the nadir PSA level [45]. Patients free from CRPC are censored at their last follow-up.
- ◦ Biochemical relapse-free survival (bRFS) will be determined from the last day of the first SBRT or from the day surgery was performed until the first day of BCR. BCR is defined as three consecutive PSA rises (1 week interval), of which at least 2 rises with a PSA level of > 2 ng/ml and a rise of 50% above the nadir PSA level. Patients free from BCR are censored at their last follow-up.
- ◦ Clinical progression free survival (cPFS) will be determined from the last day of the first SBRT or from the day surgery was performed until the first day of clinical relapse. Patients free from clinical relapse are censored at their last follow-up. Clinical relapse is defined as the appearance of 1 new lesion with a high suspicion on PSMA PET-CT/MRI [46].
- ◦ Cancer Specific Survival (CSS) will be determined from last day of the first SBRT or from the day surgery was performed until PCa death. Death of other causes is considered a competing event, while patients alive are censored at last follow-up.
- ◦ Overall Survival (OS) will be determined from last day of the first SBRT or from the day metastasectomy was performed until death from any cause, with censoring of patients alive at last follow-up.
- ◦ Acute and late toxicity score as a result of RT will be scores using the Common Toxicity Criteria Version 5.0 [47]. Toxicity will be scored at every visit during follow-up (see Table 1).
- ◦ Quality of life scoring using the EORTC QLQ-C30 supplement with QLQ-PR25. We will assess the quality-of-life-years with the EuroQOL classification system (EQ-5D-5L). The questionnaires are administered at baseline, last day of treatment, and during follow-up consultation at month M1, M3, M6, M12 and M24 (see Table 1).

### Statistical analysis

#### Sample size

The required sample size was based on the following considerations. We assume a delay of PMFS after MDT alone of approximately 21 months [9]. We consider an additional median delay of PMFS of 12 months in arm B and another additional 12 months of PMFS in Arm C as clinically meaningful. The sample size calculation was determined for a two-sided long-rank test for each of the three pairwise comparisons (Arm A vs. Arm B, Arm A vs. Arm C, Arm B vs. Arm C). We suppose a 5-years of accrual and a minimum of 5 years of follow-up to determine the primary endpoint with a 10% 5-year censoring (drop-out) rate. To account for multiple testing, a Bonferroni-correction was applied, implying a test-wise significance level of 0.0167, in order to keep a family-wise significance level of 5%. A sample size of 873 patients is required (291 patients in each arm) to obtain a power of at least 80% in each of the three tests.

#### Data analysis

Patients will be analysed in the groups to which they are assigned (intention-to-treat). A stratified two-sided log-rank test will be used for the three pairwise comparisons of the primary analysis, to test for a difference between the three treatment arms with regards to poly-metastasis free survival (PMFS). A Bonferroni correction will be applied to guarantee a study-wise 5% significance level for the primary endpoint. Stratification factors are PSA doubling time (≤ 3 vs. > 3 months), number of metastases (1 versus > 1) and initial localization of metastases (M1a vs. M1b and/or M1c). Kaplan–Meier estimates will be obtained, and a KM curve will be constructed. The hazard ratio (HR) will be determined with 95% confidence interval by means of a Cox proportional hazards model. For the evaluation of mCRPC-FS, cPFS and OS the same method will be used as for the primary endpoint. CSS will be estimated using the cumulative incidence function rather than the Kaplan–Meier estimates to count for death of other causes as a competing event.

Group differences with regards to longitudinally measured ordinal outcomes such as toxicity will be analysed by means of proportional odds models with estimation based on generalized estimating equations (GEE). Multiple imputation will be applied in case of considerable drop-out, providing consistent estimates under a missing-at-random (MAR) drop-out pattern, hence when the probability of drop-out depends on treatment group or previous observations. Group differences with regards to longitudinally measured continuous outcomes such as quality of life scores will be analysed by means of linear mixed models. Likelihood methods will then be used for parameter estimation providing valid inference under MAR. For all longitudinal analyses, the explanatory model will contain both treatment group, time point of measurement and their interaction in order to test post-hoc group effects at specific time points. Statistical analysis will be performed using SAS software.

#### Interim analysis

An interim analysis is planned approximately after 60 months and after including 179 patients in each arm. A that time we have at least 80% power to analyse arm A vs. arm B and arm A vs. arm C. To control the type I error, both tests will be evaluated at a significance level of 0.0083. If both tests are significant, arm A can be stopped. Otherwise, all arms will be continued. In such case, the final analysis will compare arms B and C at a significance level of 0.0167. In case of significance of either arm B or C versus arm A at interim analysis, the final analysis of the remaining comparisons will also be performed at a significance level of 0.0167. In case both comparisons at the interim analysis are non-significant, both arm B and C will be compared with arm A at the final analysis at a significance level of 0.0083.

#### Recruitment

Patients will be recruited via the multidisciplinary urology-oncology team meetings at the participating centres. Patients will also be included if the referring urologist agrees that this study is presented to their patients.

### Monitoring

All research physicians and research centres meet the qualification requirements of the responsible Ethics Committee in terms of professional training, experience in clinical trials and equipment. Because the trial medication is well characterized and of a low risk, no Data Monitoring Committee has been set up. Investigators will seek information on the occurrence of safety events at each participant contact. All events, whether reported by the participant or noted by Trial staff, will be recorded in a timely manner in the participant’s medical record and in the eCRF. The following minimum information will be recorded for each event: event description, start and stop date of the event, severity, seriousness, causality assessment to the IMP and/or Trial procedures, outcome. The Investigator will grant direct access to research data and documents for the purpose of monitoring, audits and/or inspections by authorized entities. As such eCRFs, source records and other study-related documentation (e.g. Investigator Site File, the Trial Master File, pharmacy records, etc.) will be kept current, complete and accurate at all times. All protocol modifications will be registered in the clinical trial protocol history. Protocol amendments will be submitted in writing to the responsible Ethics Committee and the national competent authority. All serious adverse events (SAEs) must be reported by reported within 24 h of the Investigator’s first awareness of the information. This is also the case for previously non-serious AEs which subsequently become SAEs. If the Investigator or research team becomes aware of an SAE with suspected causal relationship to the IMP or experiment, after the participant has completed the Trial, this SAE must be reported within the same timelines as for SAEs occurring during the Trial.

## Conclusion

This protocol reports the design of a randomized phase III trial to evaluate the effectiveness of the addition of short-term ADT during 1 month or during 6 months together with an androgen receptor targeted therapy (ARTA) to MDT significantly prolongs PMFS, in patients with hormone sensitive oligorecurrent prostate cancer.

## Data Availability

The datasets generated and/or analysed during the current study are not publicly available due to limitations of national privacy policies (they contain information that could compromise the privacy of the study participants) but are available on reasonable request from the corresponding author.

## Abbreviations

AAPM: American association of physicists in medicine
AE: Adverse event
ADT: Androgen deprivation therapy
ARTA: Androgen receptor targeted agents
BED: Biologically equivalent dose
BCR: Biochemical recurrence
bRFS: Biochemical relapse-free survival
CI: Coordinating investigator
cPFS: Clinical progression-free survival
CSS: Cancer specific survival
CTCAE: Common toxicity criteria of adverse effects
eCRF: Electronic case report form
GEE: Generalized estimating equations
GTV: Gross target volume
LUH: Leuven university hospitals
IMAT: Intensity-modulated arc therapy
MAR: Missing at random
mCRPC-FS: Metastatic castration-refractory prostate cancer free survival
MDT: Metastasis-directed therapy
OAR: Organ at risk
OS: Overall survival
PET-MRI: Positron emission tomography/magnetic resonance imaging
PCa: Prostate cancer
PCSS: Prostate cancer specific survival
PMFS: Polymetastatic-free survival
PSA: Prostate specific antigen
PSMA PET-CT: Prostate-specific membrane antigen positron emission tomography-computed tomography
PTV: Planning targeted therapy
QOL: Quality of life
rPFS: Radiographic progression-free survival
RT: Radiotherapy
SAE: Serious adverse event
SBRT: Stereotactic body radiation therapy
VMAT: Volumetric arc therapy
WHO: World health organization
